# Markers of Cardiovascular Risk in Postmenopausal Women with Type 2 Diabetes Are Improved by the Daily Consumption of Almonds or Sunflower Kernels: A Feeding Study

**DOI:** 10.5402/2013/626414

**Published:** 2012-12-19

**Authors:** Korina Richmond, Sheila Williams, Jim Mann, Rachel Brown, Alexandra Chisholm

**Affiliations:** ^1^Department of Human Nutrition, University of Otago, P.O. Box 56, Dunedin 9054, New Zealand; ^2^Department of Preventive and Social Medicine, University of Otago, P.O. Box 913, Dunedin 9054, New Zealand

## Abstract

Dietary guidelines for the treatment of type 2 diabetes advocate the regular consumption of nuts and seeds. Key lipid abnormalities associated with diabetes include raised LDL-C, VLDL-C, and TAG concentrations and decreased concentrations of HDL-C. The fatty acid profiles of nuts and seeds differ and may potentially influence lipid outcomes in people with diabetes differently. To examine the effects of nut or seed consumption on lipid and lipoprotein markers of cardiovascular disease (CVD), we added almonds (AD) or sunflower kernels (SKD) to a recommended diet in a randomised crossover feeding study. Twenty-two postmenopausal women with type 2 diabetes consumed personalised diets, with the addition of 30 g/d of either almonds or sunflower kernels. All food was supplied for two periods of three weeks, separated by a four-week washout. There was a significant reduction in high-density lipoprotein cholesterol (HDL-C), triacylglycerol (TAG), and apolipoprotein (apo) A1 and B100 on the SKD compared to the AD. Total (TC) and low density lipoprotein cholesterol (LDL-C) decreased significantly on both diets from baseline, with no difference between diets. A diet with the addition of either almonds or sunflower kernels has clinically beneficial effects on lipid- and lipoprotein-mediated CVD risk.

## 1. Introduction

Type 2 diabetes is a major independent risk factor for CVD, and this increased risk is partly attributed to abnormalities in lipid and lipoprotein metabolism as a consequence of hyperglycaemia and insulin resistance [1–4]. People with diabetes have a greater than twofold increase in the risk of CVD [5], and this risk is even greater in women who have an estimated fivefold higher cardiovascular mortality compared to women without diabetes [6]. Additionally, this risk is higher after menopause [7, 8], and with an ageing population the burden of diabetes is likely to increase.

The key lipid abnormalities associated with diabetes include raised LDL-C, VLDL-C, and TAG concentrations and decreased concentrations of HDL-C [1, 3, 4, 9]. Dietary intervention plays a major role in the treatment of both type 2 diabetes and dyslipidaemia with particular emphasis on the fatty acid composition of the diet as a determinant of CVD risk [9, 10].

Nuts and seeds are rich sources of *cis*-unsaturated fatty acids, and their inclusion into the diets of people with diabetes is recommended in dietary guidelines [11, 12]. There is strong evidence from both epidemiological and intervention studies for the benefits of incorporating nuts into the diet for CVD prevention and treatment of raised total and LDL cholesterol [13–21]. These studies have primarily focused on the general population and persons at high risk of CVD, not specifically due to diabetes. However one prospective study in women with diabetes reported a significant reduction in LDL-C and a 44% reduction in the risk of developing CVD in women consuming 5 or more serves of nuts per week compared to those who almost never consume nuts [22]. Only a small number of intervention studies have examined the inclusion of nuts in the diets of persons with type 2 diabetes, and these have produced inconsistent results [23–27]. While two studies have reported reductions in LDL-C when compared to control diets [23, 27], others have only shown reductions compared to baseline with no difference when compared to control diets [24–26]. Similarly, HDL increased in one study [27], but decreased in another [24]. Blood triglyceride concentrations have been relatively unaffected by the inclusion of nuts in the diet of those with diabetes [23–27]. Limitations with these studies include relatively low initial concentrations of LDL-C [24, 26, 27], high dropout rates, or weight loss which may have confounded the results [25]. Therefore to date there is limited evidence for a link between nut consumption and improved blood lipid profiles in people with type 2 diabetes.

Although studies in the general population have reported reductions in cardiovascular risk factors with the consumption of seeds [28, 29], to date there have been no intervention studies examining the incorporation of seeds into the diet of people with diabetes. The predominant fatty acids found in seeds are polyunsaturated fatty acids (PUFAs). Previous research suggests that PUFA-rich diets are associated with greater reductions in LDL-C, triglyceride, and HDL-C compared to monounsaturated-fatty-acid-(MUFA-) enriched diets. Given the predominant fatty acids in most nuts are MUFA, it is hypothesised that the consumption of seeds might influence different cardiovascular outcomes in comparison to nuts. These differences may be important in addressing a number of key lipid abnormalities associated with type 2 diabetes. To investigate this we supplied personalised diets based on the New Zealand [12] and European guidelines [11] supplemented with either almonds or sunflower seeds to postmenopausal women with type 2 diabetes, to assess the effects on CVD-mediated risk factors and glycaemic control.

## 2. Materials and Methods

### 2.1. Participants

Twenty-two postmenopausal women (mean age 62 ± 5.7 years) with type 2 diabetes were recruited into the study from General Practitioners (GPs) practices in the Dunedin area, the Diabetes Clinic at Dunedin Hospital, South Link Health and advertisements in local newspapers. Inclusion criteria were postmenopausal status and type 2 diabetes mellitus as diagnosed by the women's medical practitioners according to established criteria. The participants also needed to be able to report to the metabolic facility in the Department of Human Nutrition, University of Otago at midday from Monday to Friday during the dietary intervention periods. Exclusion criteria were current use or use within the previous year of insulin, lipid-lowering drugs or hormone replacement therapy, present smoking, allergies to nuts or seeds, or a TC above 8 mmol/L. All participants gave written informed consent, and the study was approved by the Otago Ethics Committee.

### 2.2. Experimental Design

This study utilised a randomised crossover design with two three-week interventions (AD and SKD) separated by a four-week washout when the participants returned to their habitual diets. Two blood samples were collected by venepuncture 48 hours apart after an overnight fast, at the beginning and in the final week of each intervention period. Lipids, lipoproteins, apolipoproteins A and B, oxidised LDL, insulin, vitamin E, glucose, and glycosylated haemoglobin (HbA1c) were measured. Height was measured at the beginning of the study to the nearest millimetre using a stadiometer. Participants were weighed, to the nearest 0.01 kilogram on calibrated electronic scales (Wedderburn Scales, Auckland, New Zealand), at each blood draw and weekly during the intervention periods. The same set of scales were used throughout the duration of the study. Blood pressure was measured after a 5-minute rest at baseline and at the end of each dietary treatment using a random zero sphygmomanometer (Hawksley & Sons Ltd, Lancing, UK). Participants were asked to maintain their usual level of physical activity during the course of the study.

### 2.3. Dietary Intervention

A four-day weighed diet record (4DDR) and a prestudy interview formed the basis for the personalised diets. Participants were asked to complete a 4DDR before the start of the dietary intervention and during the washout phase prior to the second dietary phase to assess habitual dietary intake. They were supplied with electronic scales (Salter Electronics, Salter Housewares Ltd., Kent, UK) accurate to 2 g, a measuring cup, and measuring spoons and given detailed verbal and written instructions on the method of completing an accurate food record. After completion of the baseline diet record the participants were individually interviewed to clarify any issues raised by the written records and to ascertain their likes and dislikes. Personalised diets to be provided during the dietary intervention were matched to each subject's habitual portion size intake to assist with the maintenance of a stable weight. The diet records were analysed using the computer programme Diet Cruncher for Macintosh (Marshall-Seeley, version 1.2.0, 2001), which utilises food composition data from the Concise New Zealand Food Composition Tables [30].

The study diet was a recommended dietary pattern for persons with type 2 diabetes based on the European and New Zealand evidence-based guidelines [11, 12]. The diets were designed to provide 30% total energy from fat of which 8% was to come from saturated fat, 15% or 9% from monounsaturated fat, and 6% or 12% from polyunsaturated fat in the AD and SKD diets, respectively. Carbohydrate and protein were to provide 51% and 17% total energy, respectively. The dietary fibre target was 25–35 g and dietary cholesterol ≤200 mg per day.

A three-week menu cycle was developed incorporating the recommended daily dietary pattern with two or more portions of fruit and three or more portions of vegetables, six portions of breads and cereals predominantly wholegrain, two portions of low fat milk and dairy products, and one portion of lean meat, poultry, fish, or eggs. Fish and a vegetarian dish were each incorporated in the main meal once every week. All meals were prepared with minimal fat, salt, and sugar, and any snacks or preprepared foods used were also low in fat, salt, and sugar. Thirty grams of whole natural almonds or 30 g sunflower kernels were added to the AD or SKD diets, respectively, each day of the three-week intervention period. From Monday to Friday they were consumed as part of the midday meal.

Almonds and sunflower kernels were chosen as they have a similar amount of saturated fat per 100 g and similar total amounts of unsaturated fats, almonds being rich in monounsaturated fat and sunflower kernels in polyunsaturated fat. The small amounts of added fats and oils needed for spreading, baking, or cooking reflected the composition of the almonds and sunflower kernels. A predominantly monounsaturated spread (Olivio, Unilever, Auckland, New Zealand) and olive oil (Lupi Extra Virgin, William Aitken & Co., Auckland, New Zealand) were used in the almond diet and a predominantly polyunsaturated spread (Meadow Lea, Goodman Fielder, Auckland, New Zealand) and grape seed oil (Azalea, William Aitken & Co., Auckland, New Zealand) in the sunflower kernel diet. Thus the nutrient composition of the AD and SKD was planned to be essentially the same apart from the differing fatty acid composition.

Participants reported to the metabolic facility at midday during the week to consume the dinner meal and take home the remaining meal items for the following 24 hours. On Fridays, participants were supplied with their entire food requirements for the weekend up until midday Monday. Beverages were not provided as part of the diet distributed by the metabolic facility but the participants kept a daily record of what they drank each day. Participants were encouraged to consume only food provided by the metabolic facility. However, a list of suitable snack foods was given to each participant to choose from if required, and they were instructed to weigh and record these foods. Electronic scales, measuring cups, and measuring spoons were given to each participant to keep throughout the dietary intervention to assist with the measurement of beverages or extra foods consumed. Furthermore, participants were asked to weigh and record any food from the metabolic facility that was not consumed. During the washout period the participants were counseled to return to the diet, which they had consumed prior to the study, and to avoid eating nuts and nut products or seeds and seed products.

### 2.4. Laboratory Methods

All blood samples were drawn from a forearm vein after a 12-hour overnight fast. Research nurses carried out all venepuncture. Blood samples were collected into Vacutainers (Belton Dickinson Diagnostics) containing dipotassium EDTA (for analysis of lipids, lipoprotein, and *α*-tocopherol), sodium fluoride (for analysis of HbA1c and glucose), and tubes containing no additives (for analysis of insulin). The duplicate blood tests were drawn approximately 48 hours apart to account for intraindividual variation in blood cholesterol concentration. Once plasma/serum and red blood cells were separated, aliquots were stored at −80°C until laboratory analyses were conducted. Plasma TC, HDL-C, TAG, glucose, and HbA1c concentrations were measured by enzymatic methods using kits and calibrators supplied by Roche Diagnostics (Mannheim, Germany) on a Cobas Mira Plus Analyser. HDL-C was measured in the supernatant following precipitation of apoprotein B containing lipoproteins with phosphotungstate-magnesium chloride solution [31]. Coefficient of variation (CV) for measurement of TC was 1.6%, HDL-C 2.8%, TAG 4.5%, glucose 1.8%, and HbA1c 1.5%. Plasma LDL-C concentration was calculated using the Friedewald formula [32]. Apolipoproteins A1 and B100 and *α*-tocopherol concentrations were measured on one of the two nonconsecutive blood samples during each period. Apolipoprotein A1 and B100 concentrations were determined by immunoturbidimetry using commercial kits from Roche Diagnostics (Mannheim, Germany). The CV for measurement of apo A1 was 3.9% and apo B100 was 2.4%. Plasma *α*-tocopherol was determined using the Agilent high-performance liquid chromatography system (1100 series) based on methods described by Thurnham et al. [33]. The CV for *α*-tocopherol was 2.4%. Serum insulin was measured by a solid-phase radioimmunoassay using Coat-a-Count kits from Diagnostic Products Corporation (Los Angeles, United States). Plasma oxidised low-density lipoprotein (oxLDL) was determined by using the oxidised LDL enzyme-linked immunosorbent assay (ELISA) kit from Mercodia (Uppsala, Sweden). Calibration and quality control were maintained by participation in the Royal Australasian College of Pathologists Association Quality Assurance Program.

### 2.5. Statistical Analysis

A sample size of 22 women was calculated to have an 80% chance of detecting a difference of 0.36 mmol/L in LDL cholesterol at the 5% level of significance. The study was primarily set up to investigate the difference in change in variables between the two diet treatments. This was analysed using a mixed model, which included a term for period and takes into account the underlying correlation between the repeated measures. These results were adjusted for baseline and order effects. The variance in change between the diets is presented as mean and 95% confidence intervals. Due to glucose, insulin, vitamin E, and oxidised LDL data being skewed, the results required logarithmic transformation. Thus the results are presented as the ratio of the geometric means with 95% confidence intervals. A *P* value of less than 0.05 was regarded as statistically significant. Further analyses were conducted to investigate the effect of each diet by using a paired *t*-test to analyse the difference between baseline and final values. Statistical analyses were performed on dietary data using computer software SPSS 11.0.2 (SPSS for Mac OS X, 2003, SPSS Incorporated).

## 3. Results 

A total of 22 women were recruited and completed both the almond and sunflower kernel dietary interventions.

### 3.1. Dietary Intake

The data from the 4DDR completed by each participant before each dietary intervention was pooled. Furthermore, a diet record was completed for each participant, each day of both diet interventions, and data pooled for the AD and SKD and presented as mean values. There were no significant differences in total energy, protein, carbohydrate, and cholesterol levels between the diets (Table 1).

When the AD was compared to the SKD, there was no significant difference in the total fat content which provided 30.3% and 30.4% total energy from fat sources, respectively. Both AD and SKD diets contributed a similar amount of SAFA 8.0% and 7.8%, respectively; however as intended the AD had almost twice the amount of MUFA compared to the SKD, 15% versus 8.9% (*P* < 0.001). The reverse was true with respect to PUFA with the SKD providing 12.5% total energy compared to only 6.3% total energy in the AD (*P* < 0.001), which was the desired target.

Fibre and vitamin E intakes were significantly different between the diets (0.028, *P* < 0.001, resp.). 

When compared to the habitual diet, the AD had significant reductions in energy, total fat, SAFA and cholesterol (all *P* < 0.001), and protein (*P* < 0.01). Fibre and vitamin E intakes were higher in AD compared to baseline (both *P* < 0.001). On the SKD there were significant reductions in energy, protein, and total fat (all *P* < 0.01) and SAFA, MUFA, and cholesterol (all *P* < 0.001) intakes when compared to the habitual diet. In comparison there were significant increases in PUFA and vitamin E (*P* < 0.001), fibre (*P* < 0.01), and carbohydrate (*P* < 0.05) intakes.

Nutrient contribution of the whole almonds and sunflower kernels to the respective diets was very similar. One serve of either whole almonds (30 grams) or sunflower kernels (30 grams) per day contributed 11% of total daily energy and provided 15.7 grams and 14.9 grams of fat per day, respectively, which equated to approximately one-third of the total fat content of the diet.

### 3.2. Lipids and Lipoproteins

There were significant differences between the dietary treatments for HDL-C, TAG, apo A1, and apo B100. The AD diet essentially maintained baseline HDL-C concentrations with a drop of 0.01 mmol/L, whereas there was a significant 0.07 mmol/L reduction (*P* = 0.050) observed on the SKD. A 12% reduction in TAG concentrations was observed on the SKD (*P* = 0.001), whereas no significant reduction was seen on the AD (*P* = 0.798). The SKD reduced apo A1 concentrations to a greater extent than the AD diet (*P* = 0.001). Similar to apo A1 results, the reduction in apo B100 concentrations was also significantly greater on SKD compared to AD (*P* < 0.001).

There were significant reductions in TC (5.6% AD and 9% SKD), LDL-C (7.4% AD and 9.5% SKD), Apo A1 (2.8% AD and 6.9% SKD), ApoB (4.8% AD and 10% SKD), and oxLDL (7% AD and 12% SKD) on both dietary treatments (Table 2).

### 3.3. Blood Pressure

Overall there were no differences in the change of SBP (*P* = 0.630) and DBP (*P* = 0.719) between the two dietary treatments (Table 2). However there was a drop in both SBP and DBP levels from baseline to the end of the two dietary treatments. The decrease in DBP from baseline was significant on both diets AD (4.6%, *P* = 0.020) and SKD (3.4%, *P* = 0.004). However the drop in SBP from baseline was only significant on the SKD (5.0%, *P* = 0.016).

### 3.4. Glycaemic Control

There was no significant difference in changes in blood glucose concentrations between the two dietary treatments (Table 3). However 10% (*P* = 0.006) and 11% (*P* = 0.002) reductions in blood glucose concentrations from baseline to end of treatment were observed on the AD and SKD, respectively. A significant reduction in HbA1c was also found on both diets (2.6%, *P* = 0.020 for AD and 3.3%  *P* = 0.017 for SKD) with no significant difference between dietary treatments (*P* = 0.091). There were no differences in the change in insulin observed on the two dietary treatments. However when compared to baseline, a significant reduction (*P* = 0.010) was observed on the SKD diet.

### 3.5. *α*-Tocopherol

The SKD caused a reduction in plasma *α*-tocopherol concentrations (4.4%), whereas the AD diet increased *α*-tocopherol (3.4%) (Table 2). The difference between the two dietary treatments was significant (*P* = 0.013).

### 3.6. Anthropometry

There was no significant difference in change in body weight between the two dietary treatments (Table 4). However a significant reduction in weight was observed when change from the start to end of each dietary phase was compared (*P* < 0.001 for AD and SKD). The mean BMI results at the start of the AD and SKD were 29.16 and 29.24 kg/m^2^, respectively. There was a reduction in BMI seen on both diets by 0.33 kg/m^2^ (AD, *P* < 0.001) and 0.32 kg/m^2^ (SKD, *P* < 0.001); however there was no difference between dietary treatments (*P* = 0.587).

## 4. Discussion

As far as we are aware this is the first study to compare the effects of almonds and sunflower kernels in people with type 2 diabetes, and it reinforces the premise that dietary intervention plays a major role in the treatment of both type 2 diabetes and dyslipidaemia. The study demonstrated clinically important improvements in CVD risk factors and glycaemic control in postmenopausal women with type 2 diabetes, who were provided with personalised diets with the addition of either almonds or sunflower kernels. In spite of this our study was limited by a small study group and short intervention period. However the cost of providing all the food for the two intervention periods of three weeks each and the considerable participant burden involved in taking part curtailed any increase in participant numbers or time. In addition the study was powered on LDL cholesterol, and the number of women and the intervention period were both calculated to be adequate to see changes in the lipid and lipoprotein end points of interest. In order to see definitive changes in HbA1c an intervention period of at least eight weeks would have been necessary.

Both diets resulted in beneficial effects on cardiovascular risk factors, namely, a reduction in TC, LDL-C, and oxidised LDL. However there were some important differences in lipid parameters between the two dietary treatments. The HDL-C concentrations were maintained on the AD, but fell on the SKD diet. Previous studies have reported reductions [24] or increases [27] in HDL-C with the addition of nuts to the diets of people with diabetes. Our study suggests that the inclusion of nuts may be beneficial in maintaining HDL-C concentrations, and it is likely that the high monounsaturated content of the almonds contributes to this effect.

A meta-analysis by Mensink and Katan [34] on fat type suggested some differences between MUFA and PUFA, favouring MUFA for beneficial effects on HDL-C. The SKD diet was predominantly PUFA, suggesting the difference in fatty acids is most likely to account for the observed difference. Hodson et al. observed a 14% reduction of HDL on a diet containing 9.1% PUFA and a 4% reduction in HDL with a predominantly MUFA (11.6%) diet [35]. The importance of HDL as a CVD protective factor at all ages and especially in women has recently been highlighted in the analysis of the SCORE dataset [36].

A further difference between dietary treatments was a reduction in TAG on the SKD compared to the AD diet. Again fat type is the most likely explanation for this finding. Studies in the general population have reported beneficial reductions in TAG concentrations on a PUFA-rich diet [35]. However, as with our study, most other studies in people with diabetes, which have included regular nut consumption, have shown no changes in TAG concentrations [23–27]. Given that low HDL-C and high TAG are often observed in people with diabetes [1, 3, 4, 9], the use of both nuts and seeds would appear to offer a sensible approach to improve these metabolic parameters. However the varieties, combinations, and amounts would need to be tested in a further intervention.

The reductions in LDL-C observed in our study are similar to other studies where nuts have been included in the diet of people with diabetes [23, 25, 27]. The significant reduction in LDL-C is likely to be due to the favourable *cis*-unsaturated fat content of both diets. Mensink and Katan [34] reported a slight beneficial effect of n-6 PUFA on reducing concentrations of LDL-C. In the present study although the reduction in LDL-C was not significantly different between the AD and SKD diets, the magnitude of the reduction was higher for the polyunsaturated-rich SKD. The cholesterol lowering properties of seeds were also demonstrated by Wu and colleagues who conducted a randomized, placebo-controlled, crossover study of two intervention periods separated by a three-week washout with 26 healthy postmenopausal women [29]. Participants consumed either 50 g/d sesame seed or the placebo of 50 g/d rice powder. Following the sesame intervention plasma total cholesterol (TC), LDL-C, and the ratio of LDL-C to HDL-C decreased by 5, 10, and 6%, respectively, which was significantly different from the placebo.

Both the AD and SKD diets were equally effective at controlling glycaemic response, with reductions in HbA1c, blood glucose, and insulin. The reductions in HbA1c of around 0.2% are relatively modest. Reductions of 0.3% have been seen in drug trials [37]. A recent trial, powered to show reductions in HbA1c, and which used intensive individualised dietary advice given to optimally medicated individuals with type 2 diabetes, reported reductions of 0.4% between intervention and usual dietary care [38]. There is a continuous relationship between complications of diabetes and HbA1c suggesting that the modest reductions observed in our study could be clinically important [39]. Fasting insulin concentrations were reduced by 17% in AD and 30% on SKD. This finding is in agreement with Tapsell et al. 2009 [26], who reported a 20% reduction with the consumption of 30 g of walnuts per day. Interestingly, Li et al. 2010 [23], reported only a 4.1% reduction in fasting insulin concentrations with the addition of 60 g of almonds. Almonds, sunflower seeds, and walnuts are all rich sources of *cis*-unsaturated fat. The addition of modest quantities of these foods appears to improve glycaemic control, with the PUFA-rich foods having a more pronounced effect.

Blood pressure was significantly reduced on both diets, with no difference between treatments. Both nuts and seeds are low in sodium and contain a variety of constituents including unsaturated fatty acids, magnesium, potassium, and other bioactive compounds, which may have beneficial effects on blood pressure. Few studies have investigated the effects of nuts on blood pressure. In the Physicians' Health Study those who consumed nuts on a daily basis had a significantly reduced risk of hypertension compared to those who did not consume nuts [40]. Estruch et al. 2006 [41], reported a reduction in both systolic and diastolic blood pressure in response to a Mediterranean-style diet containing 30 g/day nuts compared to a low fat control. A similar reduction in BP was also noted in the group consuming olive oil. This suggests that maybe the *cis*-unsaturated fat content of the diet may be playing an important role in BP reduction. In our study both nuts (rich in MUFA) and seeds (rich in PUFA) appeared equally effective at reducing BP.

Converting recommended dietary fatty acid percentages into practical food-based advice in order to reach lipid targets is a challenging part of nutrition counselling. General advice to increase unsaturated fat may inadvertently result in increased saturated fat and energy intakes. A practical approach where foods high in unsaturated fat are added to a recommended diet may be simpler and enhance compliance to dietary change. Nuts, although high in energy, have demonstrated strong dietary compensation with little change in body weight when consumed in the long term [42–45]. Therefore the addition of nuts to an optimal diet may be an appropriate approach for reaching dietary fat targets. Our study suggests that the same is true for seeds. The participants were highly motivated to adhere to their diets, and as none of them were on lipid lowering medication, this intervention gives an indication of the potential effect on lipids of diet alone. In terms of acceptability a recent study has demonstrated that the recommended amount of 30 g/d of hazelnuts, consumed over 12 weeks is both achievable and sustainable [46].

## 5. Conclusions

The addition of nuts and seeds into the diets of people with diabetes is a simple intervention that can be effective in helping to achieve the lipid targets recommended. Given the increasing rates of diabetes and the greater risk of CVD in postmenopausal women who have diabetes, it is timely to develop easily implemented dietary strategies to reduce the risk factors associated with cardiovascular disease. This study suggests that the inclusion of either nuts and seeds into a diet based on current evidence-based recommendations is beneficial and that this benefit was seen in women who were not on lipid lowering medication. Given the differential effects of nuts and seeds on HDL-C and TAG, it seems prudent to advise the inclusion of both in the diets of people with diabetes. A trial over a longer time with a larger group in a community setting, testing a combination of almonds and sunflower seeds, could be of interest.

## Figures and Tables

**Table 1:** Mean daily nutrient composition during almond and sunflower kernel diets from the 4-day diet record.

	Almond diet	Sunflower kernel diet	Difference (95% CI)	*P* value
Energy kJ	6468 (849)	6431 (885)	37 (−178, 252)	0.735
Protein g	66.1 (10.3)	67.2 (10.3)	−1.1 (−3.6, 1.5)	0.403
% total energy	17.4 (2.7)	17.8 (2.7)		
Total fat g	53.0 (14.8)	52.9 (14.9)	0.1 (−3.6, 3.7)	0.973
% total energy	30.3 (8.4)	30.4 (8.5)		
Saturated g	14 (5.2)	13.5 (5.3)	0.5 (−0.8, 1.8)	0.460
% total energy	8.0 (2.9)	7.8 (3.0)		
Monounsaturated g	26.3 (10.4)	15.5 (10.5)	10.8 (8.2, 13.4)	<0.001
% total energy	15 (5.9)	8.9 (6.0)		
Polyunsaturated g	11.0 (11.2)	21.8 (11.4)	−10.9 (−13.7, −8.1)	<0.001
% total energy	6.3 (6.4)	12.5 (6.5)		
Carbohydrate g	207.0 (32.1)	205.5 (32.5)	1.5 (−6.5, 9.5)	0.713
% total energy	51.2 (7.9)	51.1 (8.0)		
Total available sugar g	72.8 (14.9)	72.7 (14.8)	0.2 (−3.5, 3.9)	0.929
% total energy	18.1 (3.6)	18.1 (3.6)		
Cholesterol mg	135.0 (104.8)	134.8 (105.1)	0.2 (−25.9, 26.2)	0.989
Dietary fibre g	33.8 (6.3)	32.0 (6.3)	1.8 (0.2, 3.3)	0.028
Vitamin E mg	16.2 (2.1)	20.1 (2.0)	−3.8 (−4.3, −3.3)	<0.001

All values are mean (SD); *P* values are for the difference between AD and SKD.

**Table 2:** Effect of the almond and sunflower kernel diets on lipid and lipoprotein concentrations, blood pressure, and *α*-tocopherol.

	Diet	Baseline mean (SD)	Final mean (SD)	Difference (95% CI)	*P* value
Total cholesterol (mmol/L)	Almond	5.88 (0.66)	5.55 (0.65)	−0.14 (−0.29, 0.01)	0.073
	Sunflower kernel	6.11 (0.68)	5.56 (0.59)		
HDL cholesterol (mmol/L)	Almond	1.27 (0.32)	1.26 (0.31)	−0.04 (−0.085, −0.001)	0.045
	Sunflower kernel	1.32 (0.33)	1.25 (0.31)		
TC : HDL-C ratio	Almond	4.90 (1.22)	4.68 (1.26)	0.01 (−0.25, 0.26)	0.943
	Sunflower kernel	4.93 (1.41)	4.72 (1.22)		
LDL cholesterol (mmoL/L)	Almond	3.61 (0.64)	3.34 (0.64)	−0.05 (−0.19, 0.09)	0.607
	Sunflower kernel	3.77 (0.64)	3.42 (0.61)		
Triglyceride (mmoL/L)	Almond	2.10 (0.58)	2.08 (0.69)	−0.23 (−0.36, −0.09)	0.001
	Sunflower kernel	2.22 (0.71)	1.95 (0.60)		
Apo A1 (g/L)	Almond	1.41 (0.16)	1.37 (0.17)	−0.04 (−0.07, −0.02)	0.001
	Sunflower	1.45 (0.17)	1.35 (0.16)		
Apo B100 (g/L)	Almond	1.04 (0.16)	0.99 (0.17)	−0.05 (−0.08, −0.02)	<0.001
	Sunflower	1.09 (0.17)	0.98 (0.14)		
Oxidised LDL (U/L)^ †^	Almond	50.67 (35–69)	47.39 (27–65)	0.96 (0.89,1.03)^‡^	0.268
	Sunflower	52.75 (29–84)	46.93 (29–58)		
Systolic BP (mmHg)	Almond	147.36 (18.31)	141.75 (22.48)	−2.36 (−11.99, 7.25)	0.630
	Sunflower kernel	145.16 (21.05)	137.82 (20.61)		
Diastolic BP (mmHg)	Almond	88.55 (9.67)	84.50 (9.97)	0.59 (−2.60, 3.77)	0.719
	Sunflower kernel	85.93 (9.35)	83.00 (9.45)		
*α*-Tocopherol (*μ*moL/L)^†^	Almond	32.21 (17.5–94.4)	33.33 (20.7–91.5)	0.94 (0.89, 0.99)^‡^	0.013
	Sunflower	34.87 (19.2–88.4)	33.35 (19.7–63.7)		

Values are adjusted for baseline and order, ^†^results are presented as geometric mean and range, ^‡^results are presented as ratio of the geometric means, and *P* values are for the difference between AD and SKD.

**Table 3:** Effect of the almond and sunflower kernel diets on fasting concentrations of blood glucose, HbA1c, and insulin.

	Diet	Baseline mean (SD)	Final mean (SD)	Difference (95% CI)	*P* value
Glucose (mmoL/L)^†^	Almond	8.13 (4.63–15.41)	7.41 (4.87–13.12)	1.00 (0.95, 1.04)^‡^	0.851
	Sunflower	8.19 (5.03–16.48)	7.38 (4.89–15.15)		
HbA1c (%)	Almond	6.58 (1.24)	6.41 (1.20)	−0.02 (−0.20, 0.15)	0.791
	Sunflower	6.64 (1.33)	6.42 (1.27)		
Insulin (*μ*IU/mL)^†^	Almond	5.13 (1.2–30.1)	4.22 (1.0–27.4)	0.87 (0.67, 1.12)^‡^	0.276
	Sunflower	5.40 (0.9–95.2)	3.78 (0.9–16.0)		

Values are adjusted for baseline and order, ^†^results are presented as geometric mean and range, ^‡^results are presented as ratio of the geometric means, and *P* values are for the difference between AD and SKD.

**Table 4:** Effect of the almond and sunflower kernel diets on body weight and body mass index.

	Diet	Baseline mean (SD)	Final mean (SD)	Difference (95% CI)	*P* value
Weight (kg)	Almond	75.59 (15.22)	74.69 (14.49)	0.08 (−0.19, 0.37)	0.540
	Sunflower kernel	75.80 (15.88)	74.96 (15.21)		
BMI (kg/m^2^)	Almond	29.16 (5.07)	28.83 (4.83)	0.03 (−0.08, 0.14)	0.587
	Sunflower kernel	29.24 (5.29)	28.92 (5.07)		

Values are adjusted for baseline and order, and *P* values are for the difference between AD and SKD.
